# The crystal structure of a tetrahydrofolate-bound dihydrofolate reductase reveals the origin of slow product release

**DOI:** 10.1038/s42003-018-0236-y

**Published:** 2018-12-12

**Authors:** Hongnan Cao, Mu Gao, Hongyi Zhou, Jeffrey Skolnick

**Affiliations:** Center for the Study of Systems Biology, School of Biological Sciences, Georgia Institute of Technology, 950 Atlantic Drive, NW, Atlanta, GA 30332 USA

**Keywords:** X-ray crystallography, Enzyme mechanisms

## Abstract

Dihydrofolate reductase (DHFR) catalyzes the stereospecific reduction of 7,8-dihydrofolate (FH2) to (6s)-5,6,7,8-tetrahydrofolate (FH4) via hydride transfer from NADPH. The consensus *Escherichia coli* DHFR mechanism involves conformational changes between closed and occluded states occurring during the rate-limiting product release step. Although the Protein Data Bank (PDB) contains over 250 DHFR structures, the FH4 complex structure responsible for rate-limiting product release is unknown. We report to our knowledge the first crystal structure of an *E. coli*. DHFR:FH4 complex at 1.03 Å resolution showing distinct stabilizing interactions absent in FH2 or related (6R)-5,10-dideaza-FH4 complexes. We discover the time course of decay of the co-purified endogenous FH4 during crystal growth, with conversion from FH4 to FH2 occurring in 2–3 days. We also determine another occluded complex structure of *E. coli* DHFR with a slow-onset nanomolar inhibitor that contrasts with the methotrexate complex, suggesting a plausible strategy for designing DHFR antibiotics by targeting FH4 product conformations.

## Abstract (web summary)

Hongnan Cao et al. present the X-ray crystal structure of *E. coli* dihydrofolate reductase (DHFR) in complex with its reduced substrate, (6s)-5,6,7,8-tetrahydrofolate (FH4). This structure provides the first glimpse of the rate-limiting product release step of the DHFR mechanism and suggests a strategy for designing DHFR-targeting antibiotics.

## Introduction

Since its discovery in the 1950s^1–4^, the dihydrofolate reductase (DHFR, E.C.1.5.1.3) enzyme family has been a therapeutic target for cancer, infection, and autoimmune diseases^5–11^. A repertoire of Food and Drug Administration (FDA)-approved drugs acting as “antifolates” target DHFR by blocking its essential role in producing tetrahydrofolate (FH4). FH4 is a cofactor required for the synthesis of purine and thymidine nucleotides and certain amino acids by one-carbon transfer enzymes; therefore, it is an essential molecule for actively dividing cells^5–11^. As a consequence, *Escherichia coli* DHFR (eDHFR) has become a prototypical system to study enzyme dynamics and allosteric effects^12–29^, as well as the emergence of drug resistance^30–34^.

**Fig. 1:**
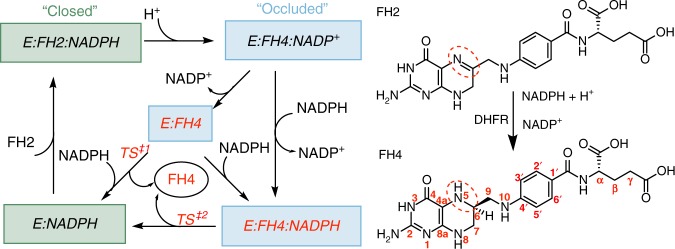
The catalytic cycle of *E. coli* DHFR

eDHFR’s catalytic cycle and molecular mechanism (Fig. 1; TS^‡1^ and TS^‡2^ denote the transition states of FH4 release pathways from the binary and ternary product complexes, respectively) have been extensively studied by various biochemical, biophysical, and computational techniques^12–29,35–38^. The chemistry of eDHFR involves hydride tunneling^39–41^ and is not rate-limiting during the steady-state catalytic cycle. A similar cycle is found for most vertebrate DHFRs despite their low, ~30%, sequence identity to eDHFR^42–44^. In contrast, as determined by primary kinetic isotope effect studies, hydride transfer is partially rate-limiting for *Lactobacillus casei* and *Streptococcus pneumonia* DHFRs^42,43^ and is predominantly rate-limiting for *Thermotoga maritima* DHFR^44^. During the process of FH2 to FH4 conversion, eDHFR cycles between closed and occluded conformational states that involve motion of the Met20 loop and nearby secondary structure elements. As evidenced by crystallography and NMR^12–29,35–38^, “closed” states only exist when the nicotinamide group of NADP(H) orients into the active site, whose entry would be otherwise blocked by the Met20 loop in the “occluded” state(s).

Over 250 X-ray, neutron and NMR structures of DHFR from various organisms and in different apo, binary, or ternary complexes with substrate, cofactor, inhibitor, and analogs have been determined and are available in the Protein Data Bank, PDB (www.rcsb.org). Among these are over 70 eDHFR structures. Despite the plethora of solved structures, the structure of a single-domain DHFR complex with the functionally mature product FH4 has never been reported. Rather, the only available models of the FH4 complexes of the single-domain DHFRs were derived from (6R)-5,10-dideazatetrahydrofolate (ddFH4) complexes^12,13,15,24,45^. Here, we have determined the crystal structure of an eDHFR:FH4 binary product complex at 1.03 Å resolution that provides an atomic snapshot of the rate-limiting product release complex. We also captured a distinct occluded conformation of eDHFR in complex with AMPQD, which to our knowledge is a novel slow-onset nanomolar inhibitor^46^. This contrasts with eDHFR’s complex with the FDA-approved antifolate drug methotrexate, where eDHFR predominantly adopts the closed conformation^24,47–49^. Both the FH4 and AMPQD complexes represent infrequent occluded conformations of eDHFR, yet they bind with nanomolar affinity and slow release of the corresponding ligands^35,36,46^. Hence, we propose a strategy to explore alternative potent inhibitors of DHFR enzymes by targeting their FH4 related, occluded conformational states.

## Results

### Isolation by crystallization of the endogenous (6s)-5,6,7,8-tetrahydrofolate-bound *E. coli* DHFR complex

**Fig. 2:**
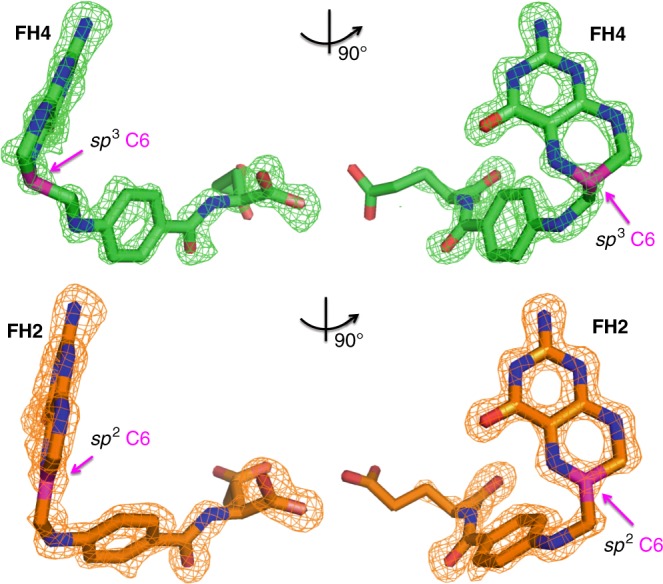
Comparison of the ligand structures of eDHFR:FH4 (green, top figure) and eDHFR:FH2 (orange, bottom figure) complexes. The Fo–Fc omit electron density maps contoured around the ligands are shown at 3.0*σ* level. The views on the right are rotated 90° counter clockwise about the vertical axis from the perspectives shown on the left. FH4 and FH2 are shown as sticks. The C6 carbon atom is colored in magenta, and its different hybridization states in FH4 and FH2 are indicated by magenta pointing arrows. All other atoms are colored as follows: oxygen in red, nitrogen in blue, carbon in green, and orange for FH4 and FH2, respectively. Only non-hydrogen atoms are shown for simplicity of viewing

The structure of the eDHFR:FH4 binary complex was determined by molecular replacement using the eDHFR:Folate:NADP^+^ closed ternary complex (PDB ID: 7DFR)^50^. As shown in Fig. 2, the clear electron density confirms the co-purified endogenous ligand as FH4 based on the tetrahedral geometry of *sp*^3^ C6 consistent with a 6s stereoisomer. This stands in contrast to the trigonal planar geometry of *sp*^2^ C6 in an FH2 binary complex obtained from similar crystallization conditions.

**Fig. 3:**
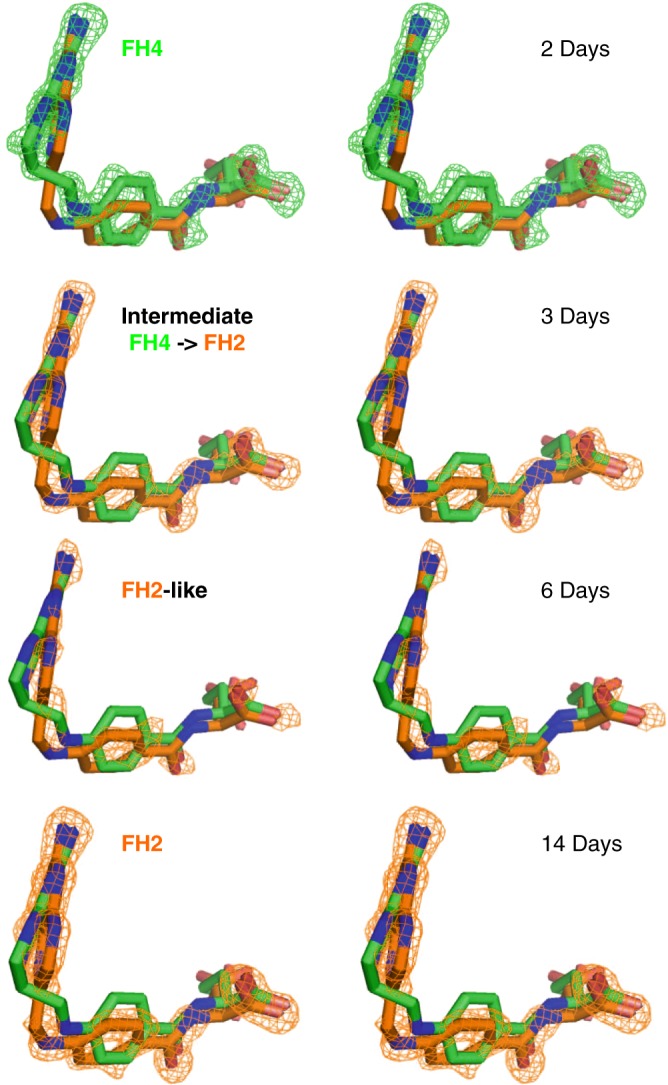
Stereo views of the time course of Fo–Fc omit electron density map changes corresponding to the conversion of FH4 to FH2. The Fo–Fc omit electron density maps contoured around the ligands are shown at 3.0*σ* level. The eDHFR:FH4 binary complex crystals were grown in the dark at room temperature. At each time point, a single crystal from an independent crystal drop was harvested by flash freezing for X-ray diffraction. The ligand structures of FH4 and FH2 of fully refined binary complex structures at 2 and 14 days, respectively, are shown in each figure as references to compare with the change of electron densities. The omit maps for the crystals harvested at 3 and 6 days were generated after initial structural refinement without introducing ligands or solvents. Superposition of the protein structures was performed using PyMOL^69^

In an effort to understand why we obtain the FH4 complex, whereas others have failed, we identified that the origin of the two different ligand complexes (FH4 vs. FH2) is the timing of crystal harvesting, and thus the duration of crystal growth. A time course study shown in Fig. 3 that follows the changes of electron densities of the bound ligand at different days of crystal growth revealed that the FH4 to FH2 decay (reflected in the *sp*^3^ to *sp*^2^ transition at C6 position) occurred approximately 2–3 days after crystallization set up.

This is the first time to our knowledge that an authentic FH4-bound single-domain DHFR complex has been isolated. We have validated the protocol to reproduce the crystallization of the eDHFR:FH4 complex and confirmed the time course of FH4 to FH2 decay by at least two independent replicates at each time point of crystal harvesting (Supplementary Fig. 1). The intermediate electron densities along the time course of the ligand’s decay clearly display the *sp*^3^ to *sp*^2^ transition at the C6 position and concomitant rotation of the benzoyl ring of the bound ligand (Fig. 3). This may resemble the transition state ligand conformation in the forward catalytic direction. The observed FH4 to FH2 decay during crystal growth likely does not reflect reverse catalysis by DHFR involving the conversion of FH4 to FH2. It is also probably not induced by light, considering that the crystallization drops were incubated at room temperature in the dark during crystal growth, and the time course of FH4 to FH2 decay is on the order of days. We have also tested co-crystallization with the reducing agents dithiothreitol (DTT) or Tris(2-carboxyethyl)phosphine (TCEP) at 2–3 mM concentration as well as introducing DTT or TCEP for up to 20 min of crystal soaking prior to harvesting at 2 days, 3 days, 14 days up to 7.5 months. Again, these procedures did not affect the reproducibility of ligand electron density changes qualitatively along the decay time course of the eDHFR:FH4 complex in the crystalline form identified in this study (Supplementary Fig. 1). Thus, it is likely that the current crystallization protocol preferentially crystallizes the endogenous FH4 complex co-purified in the eDHFR protein samples, and its decay in the crystal is irreversible under the conditions we tested, likely due to oxidation at a finite level of oxygen. Although the rapid forward catalytic reaction of producing FH4 from FH2 is thermodynamically favored in the presence of excess amount of NADPH as in vivo, the slow decay of the FH4 complex back to an FH2 complex can occur without a continued supply of NADPH as we observed here under the in vitro crystallization condition. Thus, the mystery of why the long-pursued FH4 complex was difficult to obtain is revealed to be its intrinsic instability. It is very likely the key to our success of obtaining the chemically labile FH4 complex structure is the timely harvesting of well diffracting crystals within 2 days’ growth under the crystallization condition identified here. In addition, a survey of the DHFR field indicates that many crystallographic^19,20,24,28,29,32,43,45,47,48,50–52^ and NMR^12,13,15,17,19,25,26^ studies of DHFR applied dialysis to remove endogenous ligands before introducing the exogenous ligands of interest. We identified a crystallization condition that isolates the endogenous FH4-bound DHFR complex without dialysis of the protein sample or introduction of additional substrates or products. We postulate that the current crystallization condition for eDHFR:FH4 complex favors the FH4-bound form over other forms such as the eDHFR:FH2:NADP(H) ternary complex.

### Structural characterization of the eDHFR:FH4 complex

**Fig. 4:**
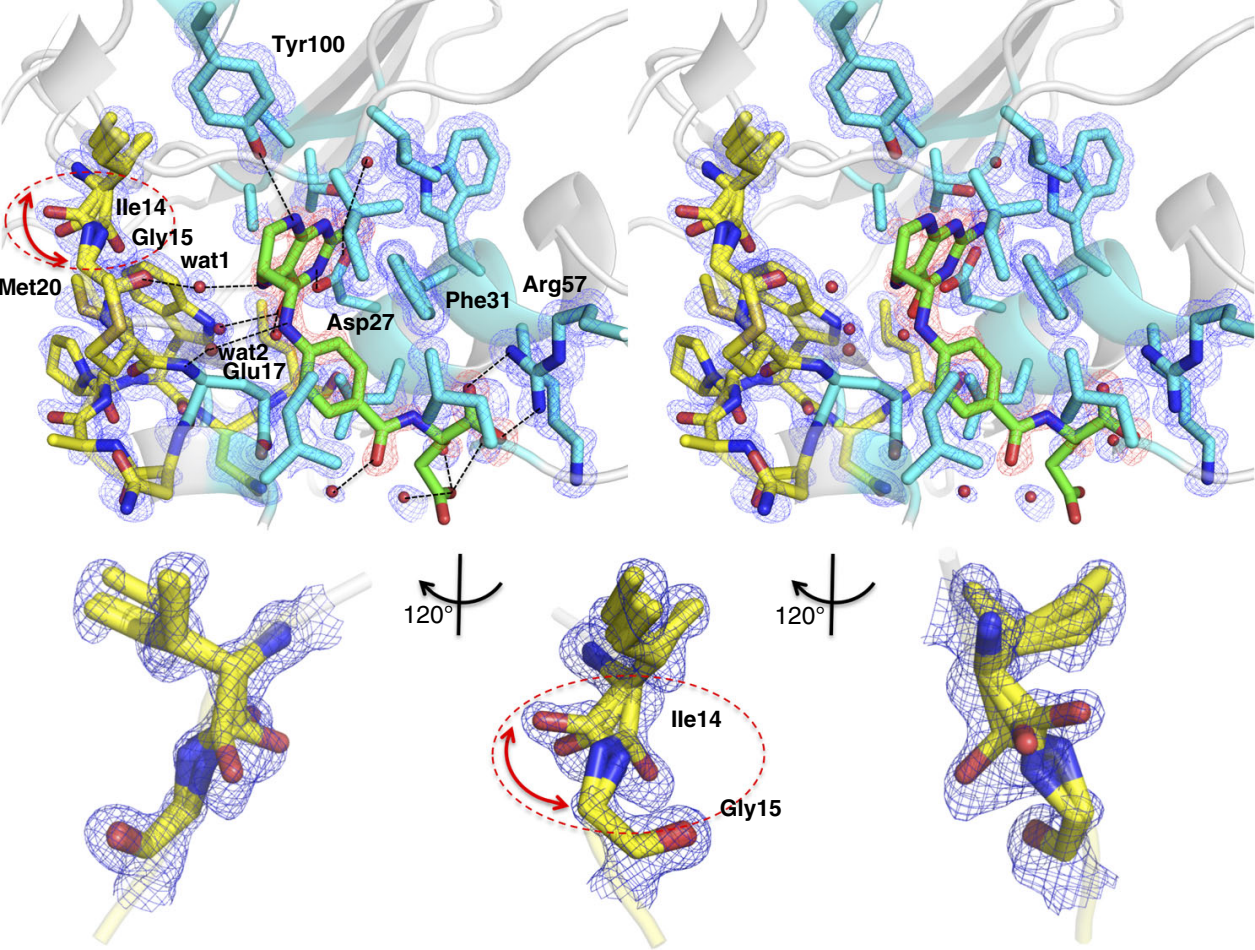
The active site structure of the eDHFR:FH4 complex shown in stereo views. **a** Side chains (cyan) within 4 Å of FH4 (green) and the Met20 loop (yellow) are shown as sticks. Secondary structures are displayed as cartoons in gray, and waters within 3.5 Å of FH4 as spheres. Polar interactions with FH4 are indicated with dashed lines. The Fo–Fc electron density map omitting FH4 is shown at a 3.5*σ* level in red and the 2Fo–Fc omit map at a 1.0*σ* level is shown in blue for residues and waters. The three conformers of Ile14-Gly15 amide linkages are indicated by a dashed circle and red arrows. **b** An expanded view of the Ile14-Gly15 linkage

**Fig. 5:**
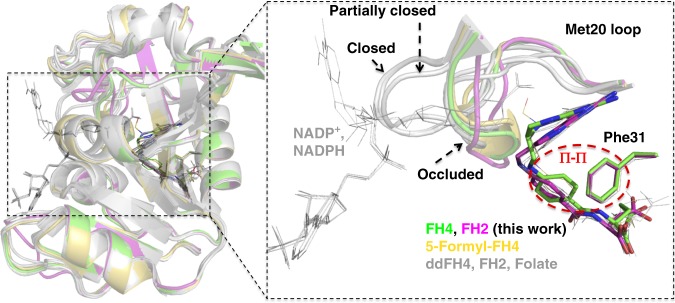
Superposition of the FH4 complex with FH2 and FH4 analog complexes. The current FH4, FH2, and reported eDHFR complexes and the reported 5-formyl-FH4 complex along with their occluded Met20 loop conformations, Phe31 residues and the corresponding ligands are colored in green, magenta, and yellow, respectively. All other structures from PDB IDs: 1DYJ (ddFH4)^45^, 5CCC (ddFH4:NADP^+^)^12^, 1RF7 (FH2)^24^, 4PDJ (FH2:NADPH)^20^, and 4PSY (folate:NADP^+^)^20^ are colored gray. The red dashed circle indicates proposed *π*–*π* interactions between Phe31 and the ligand benzoyl groups that adopt two distinct orientations depending on the bound ligands. FH4 and 5-formyl-FH4 belong to one cluster in contrast to ddFH4, FH2, and folate, while the Phe31 side chains stay in nearly the same position in all aligned structures. The Met20 loops are categorized into three general conformational states, closed, partially closed, and occluded. Only the closed conformations can structurally accommodate the nicotinamide group of the NADP(H) cofactor entering the active site

The FH4 complex adopts an occluded conformation in eDHFR (see Figs. 4 and 5). This is consistent with the previous findings that suggest that all ground state FH4 binary and ternary complexes of the catalytic cycle (post hydride transfer and *sp*^2^ to *sp*^3^ conversion at C6) occur in occluded conformations. This is due to the steric clash of the tilted pterin ring of FH4 with the nicotinamide ring of NADP(H), which would occur in the closed conformation of the Met20 loop (Fig. 5)^12–29,35–38^. As indicated in Fig. 4, FH4 has van der Waals contacts and favorable polar interactions with active site residues and waters. In particular, two bidentate salt bridges with Asp27 and Arg57 anchor the two ends of FH4, aminopyrimidine (N3 and exocyclic-NH_2_), and α-carboxylate, respectively, in near identical positions as in substrate/analog complexes^12,20,24,45^.

There are two water molecules bridging the Met20 loop and FH4 via a hydrogen bond network that involves Gly15 (C = O)-wat1-FH4(N5) and Glu17(NH)-wat2-FH4(N10) (Fig. 4). These interactions are absent in the previously reported (6R)-5,10-dideazatetrahydrofolate (ddFH4) complexes^12,45^ due to the N to C replacement at the 5 and 10 positions in the analog. This might cause the observed difference in the Met20 conformation in the analog compared to the FH4 complex (Fig. 5). The only available structures in the PDB that closely resemble the Met20 loop conformation in the FH4 complex are a 5-formyl-FH4 complex (Fig. 5, PDB ID: 1JOM)^51^ and two eDHFR-nanobody allosteric inhibitory complexes that target different DHFR epitopes with nanomolar affinity (Supplementary Fig. 2, PDB IDs: 3K74 and 4EIG)^28,29^. The 5-formyl-FH4 complex preserves the bridging water between Glu17(NH) and FH4(N10) as in the FH4 complex, despite their different space groups P6_1_ and P2_1_2_1_2_1_ respectively. 5-Formyl-FH4, also known as folinic acid or leucovorin, is an FDA-approved “rescue” drug for preventing harmful effects of methotrexate during chemotherapy^53^. The γ-carboxylic group of FH4 displays little electron density (Fig. 4), suggesting disorder or more freedom of bond rotation around the Cβ-Cγ or Cγ-Cδ *C*-axis than in other parts of the ligands.

**Fig. 6:**
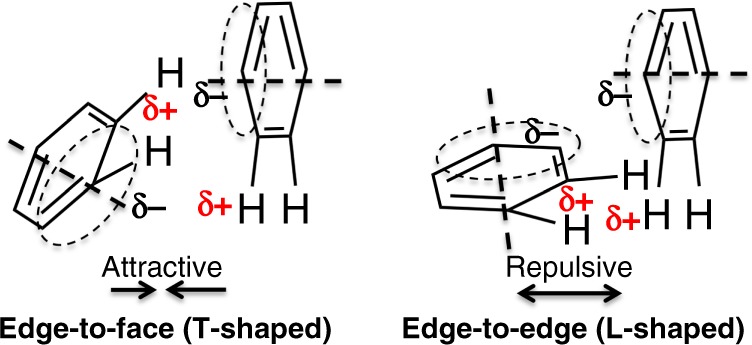
Electrostatic interactions of *π*–*π* systems. See refs ^54,55^ for details

In addition to the water network, we found the structural origin of the stabilizing interactions in the FH4 complex and slow product release based on structural comparison of current FH4 and FH2 binary complexes and previously reported eDHFR structures. Firstly, the van der Waals contact with the Glu17 side chain results in additional shielding of FH4 from solvent (Fig. 4) that is absent in substrate or product analog (6R)-5,10-dideazatetrahydrofolate complexes^12,20,24,45^. Secondly, the clear electron density of three alternative backbone conformations of the conserved Ile14-Gly15 amide linkage suggests an entropic contribution to the stability of FH4 complex from the local flexibility at the Met20 loop anchor (Fig. 4). Notably, previous mutagenesis studies showed Ile14 is crucial to control the flexibility of the Met20 loop, whereas I14V, I14A, and I14G variants all showed a slower hydride transfer rate, higher flexibility of the Met20 loop as observed in an open conformation in crystal structures, increased temperature dependence of primary kinetic isotope effect, and a higher transition state activation energy calculated from hybrid QM/MM simulations^23,40^. Thirdly, rotation of the benzoyl ring leads to electrostatically favorable edge-to-face *π*–*π* interactions with the conserved Phe31 in the FH4 complex in contrast to proton-near-proton (edge-to-edge) repulsive interactions in FH2, folate, and ddFH4 complexes regardless of NADP(H) binding (Fig. 5, expanded view, Fig. 6)^54,55^. The functional implication of this structural change is also supported by the observation of the concomitant rotation of the benzoyl ring and *sp*^3^ to *sp*^2^ transition at the C6 position of the bound ligand during the time course of FH4 to FH2 decay in the complex (Fig. 3). The role of Phe31 in controlling product release is further corroborated by previous mutagenesis studies^56^, which demonstrated that F31V and F31Y variants of eDHFR displayed a two-fold increase of the steady-state rate constant *k*_cat_ and an estimated 20- to 50-fold increase in the rate of product release in addition to the mutations’ effect on slowing down hydride transfer.

Considering the dynamic properties of the particular *E. coli* DHFR system, the stable occluded eDHFR:FH4 complex (a low free energy intermediate on its dynamic landscape) observed here may reasonably underlie the slow product release kinetics (*k*_off_ rate of FH4 dissociation, the rate-limiting step of eDHFR catalytic cycle). According to previous NMR relaxation dispersion studies, each step of the catalytic cycle of *E. coli* DHFR follows a “conformation selection” rather than “induced fit” mechanism^15^. Consequently, the microscopic rate of each step along the reaction coordinate depends on the conformational sampling rate of the enzyme^15^ (e.g. the transition state competent for rapid hydride transfer or rate-limiting product release). This implies that the more stable the ground state, and the more different it is from the excited substate, the greater the free energy cost required to sample such conformations. For eDHFR, this will necessarily involve the reorganization of the active site and the flexible Met20 loop. It was proposed in NMR relaxation dispersion studies of eDHFR^15^ that the subpopulated excited state for the hydride transfer chemical step adopts an occluded conformation (whose ground state Michaelis complex is in an closed conformation). However, the subpopulated excited state for the product release step adopts a closed conformation (whose ground state FH4 complex is in an occluded conformation). Along the reaction coordinate, the currently observed eDHFR:FH4 binary complex resides between the eDHFR:FH4:NADP^+^ and eDHFR:FH4:NADPH intermediate complexes (Fig. 1). Both adopt occluded conformations, where the nicotinamide moiety of the cofactor points away from the active site^15^. To sample the “closed excited substate” during the rate-limiting product release step^15^, active site reorganization from the occluded ground state must occur. This is represented by the stable catalytic intermediate eDHFR:FH4 captured in this study. The product dissociation rate *k*_off_ of FH4 was increased upon cofactor binding with a two-fold increase for eDHFR:FH4:NADP^+^ compared to eDHFR:FH4, and an eight-fold increase for eDHFR:FH4:NADPH compared to eDHFR:FH4 measured at both pH 6 and pH 9 by competition experiments^35^. This indicates accelerated product release and increased conformational sampling rates when a cofactor is bound. Although an authentic eDHFR:FH4:NADPH ternary complex ground state structure was never reported before, we hypothesize that there can be appreciable similarity to the eDHFR:FH4 binary complex, as all of the FH4-bound ground intermediate states adopt an occluded conformation^15^. We do expect, however, that cofactor binding will increase the population of the excited substates, proposed previously based on NMR relaxation dispersion studies to be in a closed conformation^15^. Consistent with this, we observed that in a ternary complex of the eDHFR:FH2:NADP(H) structure (also determined in our study under separate crystallization conditions), the Met20 loop became disordered. This suggests a general mechanism of cofactor facilitated ligand exchange by enhancing the conformational sampling rate when the cofactor is bound with its nicotinamide moiety pointing away from the active site.

In the FH4 complex, the distance between the FH4 benzoyl ring (C1ʹ) and the Phe31 (Cζ) is 4.93 Å, which is significantly shorter by (~0.3–0.6 Å) than the corresponding distances in current FH2 and previously reported FH2 complexes (PDB ID: 1RF7, 4PDJ)^20,24^, which are 5.22, 5.55, and 5.32 Å, respectively. A similar trend of distance shortening along the reaction coordinate of eDHFR was emphasized in two independent computational studies. A QM/MM study calculated that the corresponding distance is shortened by ~0.3 Å from the Michaelis complex to the transition state as the hydride transfer reaction occurs and that there is little difference in this distance (~0.01 Å) between the transition state and the reaction product^27^. Another study using mixed quantum/classical molecular dynamics suggested a more dramatic shortening of the corresponding distance by ~1 Å as the reaction evolves from the reactant to the transition state^18^. Therefore, our crystallographic observations are in general agreement with previous computational modeling suggesting that, to a certain extent, the FH4 complex preserves the physical nature of the transition state. This is also consistent with previous observations on the dynamic energy landscape of eDHFR mapped by NMR relaxation dispersion that each intermediate in the catalytic cycle samples low-lying excited states whose conformations resemble ground-state structures of the preceding or following intermediates^15^. Since enzymes stabilize the transition state, slow product release of the DHFR family might be attributable to the carryover of the physical nature of the transition state to the reaction product complex. This is suggested from the long-pursued FH4 complex determined here, in addition to species-specific conformational changes required during the catalytic cycle^32^.

### Characterization of an occluded complex of eDHFR with a nanomolar binding affinity slow-onset inhibitor

**Fig. 7:**
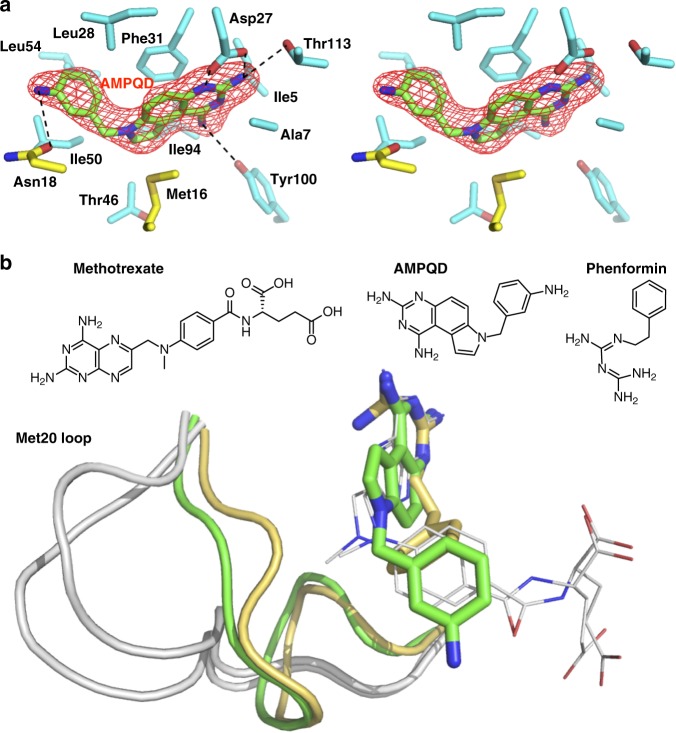
Structure of the eDHFR:AMPQD inhibitory complex. **a** Stereo view of the active site interactions with AMPQD with the Fo–Fc omit map at a 3.5*σ* level. Protein side chains (cyan) within 4 Å of AMPQD (green) are shown as sticks, including two residues from the Met20 loop (yellow). Polar interactions are indicated with dashed lines. **b** Superposition of AMPQD (green), phenformin (yellow, PDB: 5UIH)^52^, and methotrexate complexes (gray shown as thin sticks from PDB: 1RA3, 1DDS)^20,47^. Met20 loops are shown as cartoons and ligands as sticks. The ligands’ chemical structures are drawn on top. NADP(H) is not displayed in any of the structures for simplicity of viewing

X-ray crystallography shows that the complex of eDHFR with a slow-onset tight inhibitor AMPQD^46^ also displays the occluded conformation. The Met20 loop adopted a conformation in the AMPQD complex that resembles that of the ternary complex with an anti-diabetic biguanide phenformin and NADP^+^ (PDB ID: 5UIH)^52^. On the other hand, the FDA-approved chemotherapeutic agent methotrexate was previously demonstrated by X-ray crystallography^24,47^, NMR^48^, and single-molecule kinetics^49^ to bind in the closed DHFR conformation (Fig. 7). This discrepancy in protein conformations was unexpected since all three inhibitors share a common structural feature: the biguanide group of phenformin, the diaminopyrimidine group of AMPQD, and the diaminopterin group of methotrexate, each connected to a phenyl group with a flexible linker. However, a close examination from the structural superposition of the corresponding eDHFR-inhibitor complexes (Fig. 7) showed that the methylamino linkage group of the methotrexate (absent in phenformin and AMPQD) occupied a position that would result in a potential steric clash with the Met20 loop if it adopted an occluded conformation as in the phenformin and AMPQD complexes. We previously demonstrated that AMPQD displayed a relatively higher preference (a three-fold decrease in IC-50 and *K*_i_) for inhibiting eDHFR over human DHFR^46^. An even higher species-specificity for *E. coli* over human DHFR (~30-fold) is observed for the parent compound of AMPQD, which lacks the aminophenyl tail group and the methylene linker^46^. The current crystal structure of the occluded complex of eDHFR with AMPQD provides a plausible mechanistic explanation for its species-specificity, attributable to differences in conformational equilibria of human DHFR vs. eDHFR. The former is exclusively observed in closed conformations, while the latter shows higher conformational flexibility sampling in both closed and occluded conformations, as discussed next.

### Comparison of DHFR conformations based on clustering

**Fig. 8:**
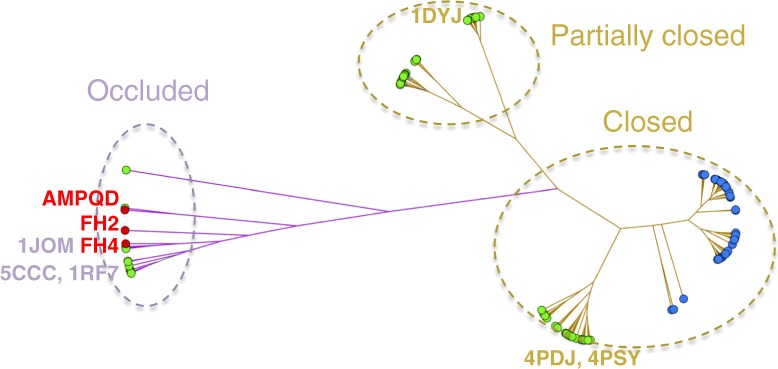
Clustering of 162 DHFR structures based on their pairwise Cα RMSD of the Met20 loops. DHFR structures are represented by circles filled in blue (humans), green (eDHFR), and red (in this study). The edge length (colored in purple for the occluded and gold for the closed conformations, respectively) is proportional to the maximum RMSD of the Met20 loop conformers. Please see a more detailed clustering diagram in the Supplementary Information

**Fig. 9:**
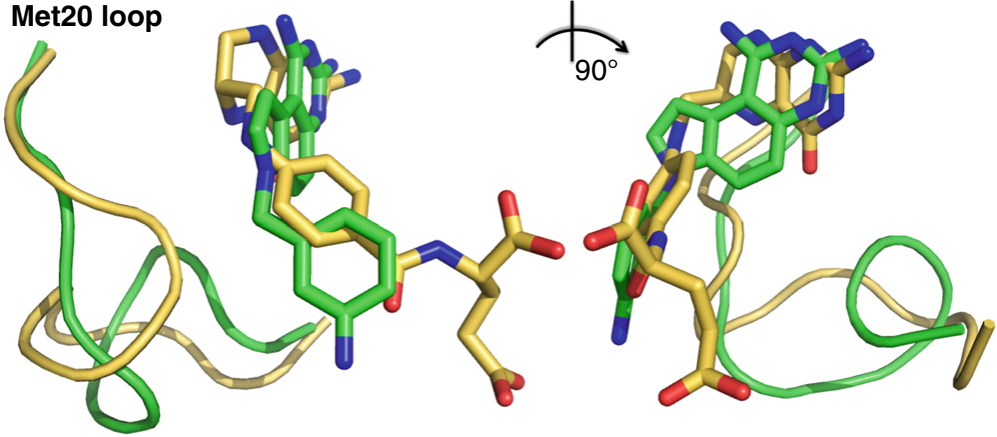
Superposition of AMPQD (green) and FH4 complexes (yellow). The Met20 loops are shown as cartoons and ligands as sticks. The view on the right is rotated 90° clockwise about the vertical axis from the perspective shown on the left

A clustering of DHFR PDB structures using the RMSD of the Met20 loop backbone Cα atoms as the distance metric (Fig. 8 and Supplementary Fig. 3) indicates that human DHFR exclusively adopts a closed conformation (catalytically competent for NADPH binding), whereas eDHFR is much more flexible with both closed and occluded conformations. The occluded conformations are less frequently seen (17%) in eDHFR structures. Both the rate-limiting product release complex with FH4 and the slow-onset inhibitory complex with AMPQD adopt an occluded conformation of eDHFR (Fig. 9) that is rarely represented in the PDB (Supplementary Fig. 3). Interestingly, both FH4 and AMPQD share the characteristics of nanomolar affinity and slow release from eDHFR^35,36,46^ with the position of the key nitrogen atoms on the heterocycles strongly conserved, and differences evident in the tails. This suggests a new strategy for developing DHFR inhibitors by targeting occluded eDHFR conformations. We also propose a strategy to combat drug resistance. As shown in Supplementary Fig. 4, on comparing the conformation of AMPQD to FH4 and trimethoprim to FH4, there are subtle differences in the van der Waals envelopes. The trimethoprim eDHFR escape variants of *E. coli* DHFR possess mutations that also block the inhibitory function of AMPQD^57^. By studying the differences in interactions, one can search for other ligands that minimize these interaction differences with FH2 and FH4. This might ensure that mutations, which diminish inhibitor binding, will also diminish the binding affinity of FH2 and FH4.

### Characterization of a ternary complex of eDHFR

**Fig. 10:**
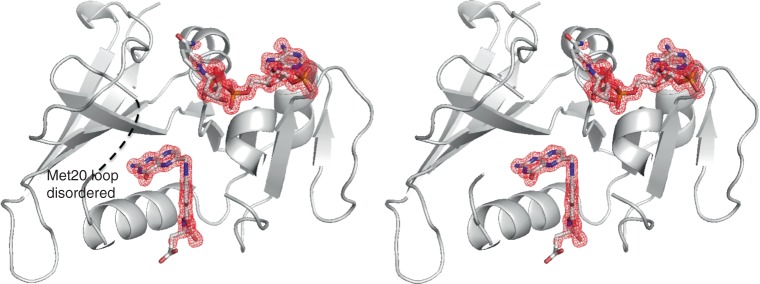
Stereo views of the DHFR:FH2:NADP(H) ternary complex. The disordered Met20 loop (residues between Ile14 and Pro21) is indicated as black dashed lines. The Fo–Fc omit map at a 3.0*σ* level is shown as red mesh. Secondary structures are shown as cartoons and ligands in a stick representation. Atoms are colored as follows: carbon (white), nitrogen (blue), oxygen (red), and phosphorous (orange)

Finally, in an eDHFR:FH2:NADP(H) ternary complex with both co-purified endogenous ligand and cofactors (Fig. 10), we found that the Met20 loop becomes disordered. This supports the role of cofactor binding in enhancing conformational sampling for rapid ligand exchange or facilitating product release via an allosteric mechanism (TS^‡2^, Fig. 1)^12–15^. The nicotinamide ribose moiety swings away from the active site (Fig. 10) similar to the occluded FH4 ternary complex^12^. Its redox state is unknown based on the electron density. The ability to isolate different endogenous ligand-bound, binary and ternary eDHFR complexes in varying crystallization conditions suggests that eDHFR contains a mixture of molecular species with different bound ligands and an ensemble of conformations. The effectiveness of the crystallographic approach applied here takes advantage of the molecular inhomogeneity by omitting the dialysis step to isolate a long-pursued and chemically labile FH4 complex crystal structure. This is opposed to the typical process involving pretreatment of DHFR samples by dialysis, which removes trace endogenous ligands and increases sample homogeneity. Improved homogeneity generally improves the overall success rate of co-crystallization or crystal soaking experiments, when the ligands of interest are exogenously introduced.

## Discussion

We have determined the structural basis for the rate-limiting product release of the eDHFR:FH4 binary complex. Analysis and comparison of the current FH4 complex with previous thermodynamic, kinetic, crystal structural, molecular dynamics, and NMR relaxation dispersion studies suggests the persistence of the ligand’s structure from the transition state to the reaction product complex. The observed Met20 loop’s conformational dynamics in the current eDHFR substrate/product/inhibitor complexes are consistent with ligand-dependent conformational sampling and energy landscape shaping during catalysis. Exploiting the conformational diversity of eDHFR, especially targeting the energetically favorable occluded conformations of the FH4 and AMPQD complexes and DHFR’s allosteric sites, may enable the design of effective next-generation therapeutics to target DHFR with species-specificity^38,57^.

During our determination of the first eDHFR:FH4 complex structure, a report appeared on the structure of a *Trypanosoma cruzi* bifunctional dihydrofolate reductase-thymidylate synthase (DHFR-TS) ternary complex with FH4 and NADP^+^ (PDB entry: 5T7O)^58^. The DHFR-TS family proteins are absent in humans, but have been extensively studied in human parasites and plants^59,58^. DHFR-TS enzymes typically contain an N-terminal DHFR fused to a C-terminal TS with the two domains that not only structurally intimately interact but also significantly affect their mutual function^58^. Comparing these two structures (Supplementary Fig. 5), there is high structural similarity in the pterin moiety (bond length RMSD of 0.31 Å) and C6 *sp*^3^ geometry (bond angle RMSD 2.3°) of the bound FH4 despite their DHFR domains’ low sequence identity of only 33.5%. We also observed a distinct difference in the orientation of the benzoyl ring of FH4 relative to the conserved Phe residue (Phe31 in eDHFR and Phe52 in *T. cruzi* DHFR-TS; Supplementary Fig. 5) suggesting a favorable *π*–*π* interaction in the eDHFR:FH4 binary complex (Fig. 6). However the Met20 loop is in the occluded conformation in eDHFR and is disordered in DHFR-TS. This structural comparison of the only two available authentic FH4 complexes of DHFRs shines light on cofactor facilitated product release mechanism via both enhanced active site conformational sampling to promote ligand exchange^12–15^ and the proposed favorable *π*–*π* interaction being disrupted upon Met20 loop disorder. This *π*–*π* interaction switch might also be functionally relevant based on the concomitant rotation of the benzoyl ring of FH4 relative to the universally conserved Phe31 in eDHFR during C6 *sp*^3^ to *sp*^2^ chemical transition when FH4 is slowly reoxidized to FH2 absent of NADPH (Fig. 3).

The notorious instability of the mature FH4 cofactor (rapid decomposition with a half-life <5 min in solution at neutral pH)^61^, and to some extent, the substrate FH2, even in isolated solid form^62^, has been a hurdle in the crystallization of the intact physiological ligand (FH4 or FH2) bound complexes of the well-studied *E. coli* and human DHFRs. Our current success in using crystallization to isolate the protein complex with the endogenously bound labile metabolite agrees with the rate-limiting product release molecular mechanism and conformational sampling of DHFR in solution. This may also suggest a multiple physiological role of DHFR. It not only produces but also protects FH4 from degradation via slow release prior to its utilization by one-carbon transfer enzymes in downstream FH4-dependent metabolism pathways. Interestingly, it has been reported that the transient interactions between eDHFR and enzymes in one-carbon metabolism pathways are fine-tuned together with the corresponding protein expression levels to maintain a balanced fitness using *E. coli*. as a model system^60^. In addition, there has also been a previous study on the stabilization of labile tetrahydrofolate by bovine milk folate-binding protein^63^.

The physiological relevance of possible FH4 in human DHFR via slow release might be even more pronounced in the acidic microenvironment of cancer where the limited extent of recycling folate metabolites for one-carbon metabolism is expected, as the free form of the mature cofactor FH4 was shown to undergo irreversible oxidative decay to pterins (without formation of FH2) at a pH below 7 (ref. ^64^) and also reducing agents was reported to no longer prevent oxidative degradation of FH4 at acidic pH^61^. On the other hand, the hypoxic tumor microenvironment might offset some of these damaging effects due to its acidic pH. This may benefit tumor survival and growth by stabilizing FH4 and possibly FH2 as well due to lack of oxygen. In conclusion, the current work has implications not only for the role of DHFR conformational variability in catalysis but also in the exploitation of this variability in the design of species-specific DHFR-based antibiotics or chemotherapeutic agents.

## Methods

### Protein expression and purification

C-terminal 6xHis-tagged *Escherichia coli* DHFR (sp|P0ABQ4|) was generously provided by Drs. Eugene Shakhnovich and João Rodrigues from Harvard University. It was overexpressed in *E. coli* BL21, and purified by Ni-NTA and size exclusion chromatography as previously described^31^. The initial protein stock was stored at −80 °C at a concentration of 30 mg ml^−1^ in 20 mM Tris, pH 8, 1 mM DTT. Polyethylene glycol 3350 and 6000 (PEG 3350 and PEG 6000) solutions were purchased from Hampton Research. AMPQD (CAS 77681-42-6 or NSC309401) was obtained from the National Cancer Institute. Its chemical structure was corrected to contain a meta rather than para aminophenyl group based on the experimental electron density in its protein complex. All other chemicals and reagents were obtained at the highest quality or purity available from Sigma-Aldrich or ThermoFisher and used without further purification.

### Crystallization and X-ray structure determination of *E. coli* DHFR

Initial crystallization trials were set up on Intelli three-well plates (Hampton Research) using the sitting drop vapor-diffusion method tested against four commercial high-throughput screens (Index, PEGRx, Crystal Screen and SaltRx from Hampton Research), each with 96 conditions, followed by optimization on MRC two-well plates (Hampton Research). The FH4 binary complex was crystallized by sitting drop vapor diffusion using a 1:1 v/v mixing of 20 mg ml^−1^ DHFR solution in 13.3 mM Tris, pH 8, 16.7 mM HEPES pH 7.3, 33.3 mM NaCl, 0.67 mM DTT with the reservoir solution containing 0.1 M MES, pH 6.5, 30% w/v PEG 3350, 0.4 M MgCl_2_. Mixed drops of 0.8 μl were equilibrated over a reservoir solution of 50 μl and incubated at 20 °C in the dark. The dihydrofolate (FH2) binary complex was crystallized using a 1:1 v/v mixing of 15 mg ml^−1^ DHFR solution in 10 mM Tris, pH 8, 25 mM HEPES pH 7.3, 25 mM NaCl, 0.5 mM DTT with the reservoir solution containing 0.1 M MES, pH 6.5, 27.5% w/v PEG 3350, 0.4 M MgCl_2_. Both FH4 and FH2 binary complex crystals appeared as a rectangular block shape after 2–5 days with the longest dimension ranging from 0.2 to 0.8 mm. The FH2:NADP(H) ternary complex was obtained using 1:1 v/v mixing of 30 mg ml^−1^ DHFR solution with 20 mM Tris pH 8, 1 mM DTT with a reservoir solution containing 0.1 M imidazole, at a pH 8 with 20% w/v PEG 6000, 50 mM calcium acetate. Crystals were long and thin plates. The AMPQD binary complex was obtained by dialysis of DHFR against 80 μM AMPQD in 50 mM HEPES, pH 7.3, 100 mM NaCl for 1 week at 4 °C in the dark using a 10 kDa cutoff Slide-A-Lyzer dialysis cassette (ThermoFisher) to remove all endogenous ligands. It was then concentrated to 8–10 mg ml^−1^. DHFR containing 80 μM AMPQD was supplemented with 0.5 mM NADPH before 1:1 v/v mixing with a reservoir solution containing 0.1 M citrate, pH 3.5, 15% PEG 6000, 150 mM lithium sulfate. Crystals were long and thick rods with longest dimension of 0.05–0.5 mm. Crystals of the FH2 and FH4 binary complexes, obtained after crystal growth for 2 weeks and 2 days, respectively, were cryoprotected by MiTeGen’s LV CryoOil (MiTeGen) and flash-frozen in liquid nitrogen. Crystals of the FH2:NADP(H) (grown for 4 weeks) and AMPQD (grown for 3 days) complexes were directly flash-frozen in liquid nitrogen without cryoprotectant. Crystals of the intermediate states between FH2 and FH4 binary complexes were grown under the same conditions as the FH4 complex and harvested at 3 and 6 days after setting up the crystallization drops, cryoprotected with LV CryoOil, and flash-frozen. The decay of the FH4 to FH2 in the crystals over a 2–3-day period was observed using electron density maps omitting the ligands. We also tested co-crystallization with the reducing agent DTT or TCEP at 2–3 mM concentration and by introducing DTT or TCEP for up to 20 min of crystal soaking prior to crystal harvesting at 2 and 3 days. These procedures did not affect the decay time course of the ligand electron density (Supplementary Fig. 1).

Diffraction data were collected at the Advanced Photon Source at Argonne National Laboratory on the LRL-CAT (31-ID-D) beamline at 100 K. The detector was a Rayonix 225 HE CCD (Rayonix) using a single wavelength of 0.97931 Å. The FH4 complex data set was collected and processed to a resolution of 1.03 Å, the FH2 binary complex to 1.11 Å, the ternary complex with FH2 and NADP(H) to 1.30 Å, and the AMPQD complex to 2.20 Å (Supplementary Table 1). The data sets were indexed, integrated, and scaled using XDS^65^. The structures were determined by molecular replacement with Phaser_MR^66^ (using the search model PDB ID: 7DFR^50^) and completed by alternating rounds of manual model building COOT^67^ and phenix.refine of the PHENIX suite^68^. The crystals of the intermediate states between FH2 and FH4 binary complexes harvested at 3 and 6 days both diffracted at LRL-CAT beamline to 1.35 Å and the corresponding models were subjected only to initial refinement after molecular replacement omitting all ligands to generate omit electron density maps to minimize bias. Using the same procedure, omit maps were computed for independent experimental replicates (*n*≥2 for each time point) to validate the reproducibility of the overall observation of the time-resolved crystallography. Each data set was collected for a single crystal at different time points of crystal harvesting ranging from 2 days up to 7.5 months (Supplementary Fig. 1). Certain time point replicates included variations of the conditions between native, soaked crystals or cocrystals with DTT or TCEP to test the effect of reducing agents (Supplementary Fig. 1). Refinement statistics are summarized in Table 1 and further details including resolution cutoff criteria are available in Supplementary Table 1. All the structures determined in this study display Ramachandran statistics absent of outliers, with 97.5–99.0% of residues in the most favored regions and 1.0–2.5% of residues in the additionally allowed regions of the Ramachandran diagram (Supplementary Table 1). All the structures were presented using PyMOL^69^. The coordinates and reflection files of the structures are deposited in the Protein Data Bank (www.rcsb.org) under PDB IDs: 6CW7 (E:FH4), 6CXK (E:FH2), 6CYV (E:FH2:NADP(H)), 6CQA (E:AMPQD).

**Table 1:** Data collection and refinement statistics

	FH4 complex^a^	FH2 complex^a^	Ternary complex^a^	AMPQD complex^a^
*Data collection*				
Space group	*P*2_1_2_1_2_1_	*P*2_1_2_1_2_1_	*P*2_1_2_1_2_1_	*P*6_1_2 2
Cell dimensions				
* a*, *b*, *c* (Å)	33.9, 51.5, 77.8	33.7, 51.5, 77.4	34.9, 58.8, 79.3	64.7, 64.7, 215.7
*α*, *β*, *γ* (°)	90.0, 90.0, 90.0	90.0, 90.0, 90.0	90.0, 90.0, 90.0	90.0, 90.0, 120.0
Resolution (Å)	42.97–1.03 (1.09–1.03)^b^	42.89–1.11 (1.18–1.11)^b^	32.87–1.30 (1.35–1.30)^b^	34.17–2.20 (2.28–2.20)^b^
*R*_sym_	0.064 (1.02)^b^	0.067 (0.86)^b^	0.085 (2.11)^b^	0.123 (2.93)^b^
*I* / *σI*	11.3 (0.7)^b^	13.4 (1.2)^b^	12.3 (0.9)^b^	19.8 (0.7)^b^
Completeness (%)	97.3 (84.2)^b^	97.9 (87.3)^b^	99.8 (99.5)^b^	98.5 (89.4)^b^
Redundancy	6.4 (2.8)^b^	6.3 (2.8)^b^	7.1 (6.3)^b^	16.6 (6.8)^b^
*Refinement*				
Resolution (Å)	31.06–1.03 (1.07–1.03)^b^	30.95–1.11 (1.15–1.11)^b^	32.87–1.30 (1.35–1.30)^b^	34.17–2.20 (2.28–2.20)^b^
No. of reflections	65,896	53,202	40,798	13,920
*R*_work_/*R*_free_	0.186/0.206	0.179/0.196	0.176/0.204	0.223/0.259
No. of atoms	1672	1637	1614	1339
Protein	1428	1394	1293	1267
Ligand/ion	39	41	80	33
Water	205	202	241	39
*B*-factors				
Protein	14.7	17.0	21.7	69.9
Ligand/ion	24.9	25.4	26.3	63.9
Water	26.3	28.6	34.7	65.5
R.m.s. deviations				
Bond lengths (Å)	0.008	0.009	0.013	0.006
Bond angles (°)	1.39	1.33	1.52	1.01

^a^Each structure was refined against a single data set from an independent protein crystal

^b^Values in parentheses are for highest-resolution shell

### Clustering-based structural analysis

Using the canonical proteins sequences for the human and *E. coli* DHFR, we searched for all crystal structures of human and *E. coli* DHFRs from the PDB. After removing those with a disordered Met20 loop with missing coordinates, we obtained 76 human and 83 *E. coli* DHFR entries. These entries and the three structures determined here were subjected to clustering. For each pair, global sequence alignments were first performed. The aligned residues were superposed by minimizing their global RMSD, and then, the RMSD of the Met20 loop’s Cα residues (residues 14–23 for *E. coli* and 16–25 for humans) were calculated. Finally, using this Met20 loop RMSD distance matrix, the standard average linkage method^70^ generated the hierarchical clustering dendrogram shown in Supplementary Fig. 3.

## Supplementary Information

Supplementary Information

## Data Availability

The atomic coordinates and structure factors are deposited in the Protein Data Bank (www.pdb.org) with ID codes 6CW7 (E:FH4), 6CXK (E:FH2), 6CYV (E:FH2:NADP(H)), and 6CQA (E:AMPQD). All other data supporting this study are available within the Article and its Supplementary Information file, or from the authors upon reasonable request.
