# Supplementary material for: The crystal structure of a tetrahydrofolate-bound dihydrofolate reductase reveals the origin of slow product release
Source: Commun Biol. 2018 Dec 12;1:226. doi: 10.1038/s42003-018-0236-y (PMC6290769; doi:10.1038/s42003-018-0236-y)
Supplement: Supplementary file 1 — Supplementary Information [file 42003_2018_236_MOESM1_ESM.pdf]

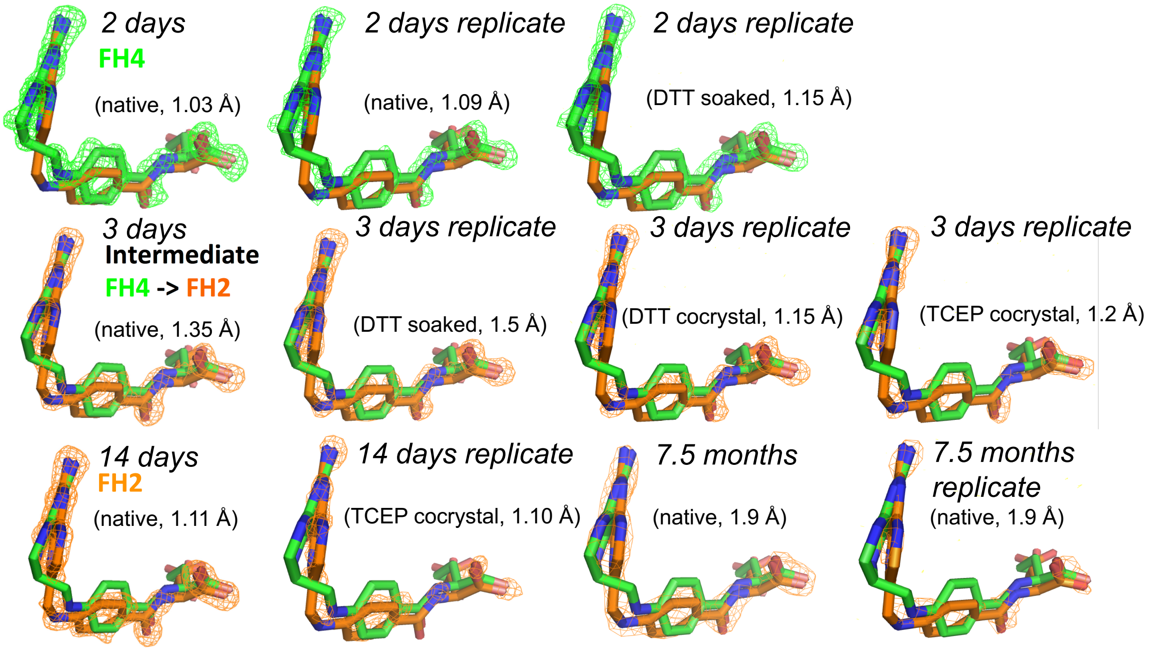

Supplementary Fig. 1, related to Fig. 3. The time course of Fo-Fc omit electron density map changes corresponding to the conversion of FH4 to FH2 in replicates. The ligand structures of FH4 (carbon in green) and FH2 (carbon in orange) of fully refined binary complex structures at 2 days and 14 days respectively are shown in each figure as references to compare with the change of electron densities. The Fo-Fc omit electron density maps contoured around the ligands are shown at 3.0 σ level. The omit maps for the crystals harvested at 3 days and 6 days were generated after initial structural refinement without introducing ligands or solvents. Superposition of the protein structures was performed using PyMOL[5]. The diffraction quality (resolution) of the crystals harvested at 7.5 months appeared to be generally poorer than fresh crystals as expected. Introducing reducing agents at 2-3 mM concentration by co-crystallization or crystal soaking for 10-20 minutes didn't affect the decay time course of the eDHFR:FH4 complex qualitatively based on electron density changes . The major conversion of FH4 to FH2 under current conditions was observed to occur as early as 2-3 days. Each time point of crystal harvesting contains at least two replicates, with the corresponding condition of reducing agents and resolution of dataset in the parentheses.

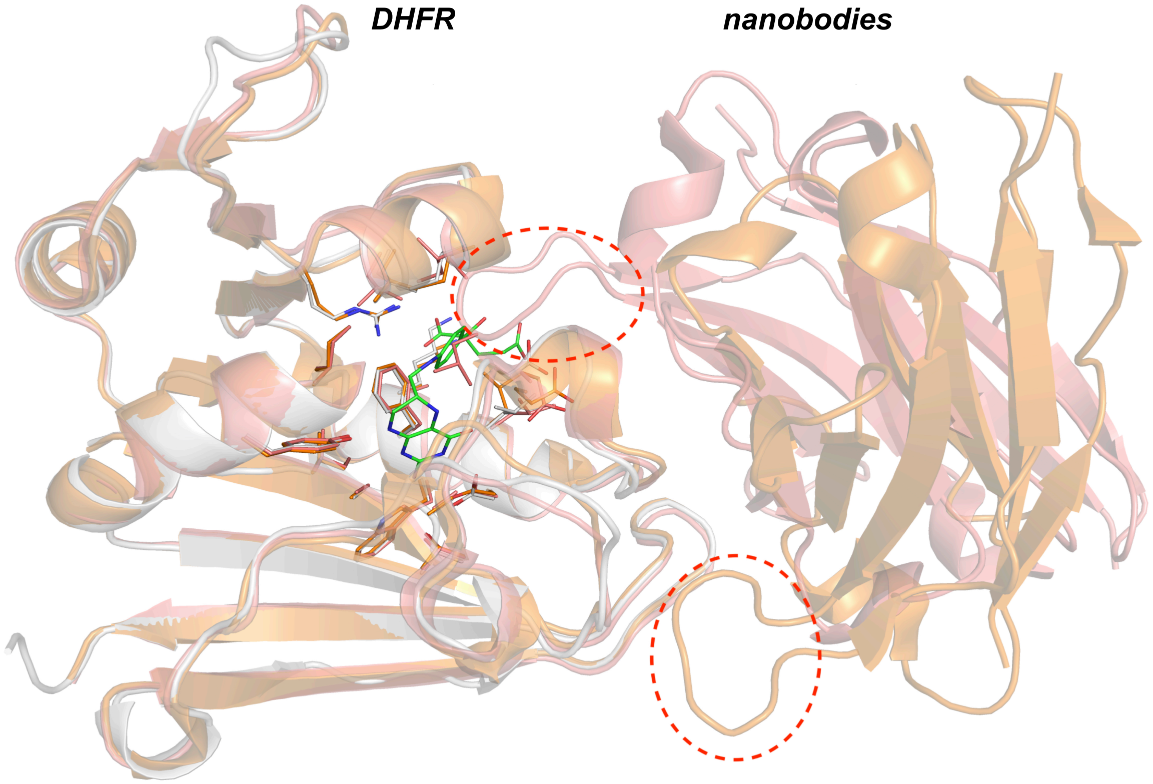

Supplementary Fig. 2. Structural similarity of the FH4 binary complex (white) and nanobody inhibitory complexes with PDB IDs: 3K74[3] (orange) and 4EIG[4] (pink). The FH4 ligand (green) and surrounding residues within 4 Å are shown as sticks in orange and pink colors for the nanobody complexes and grey for the FH4 complex. Structures were aligned by PyMOL[5] with a Cα RMSD of 0.366 for 130 residues and 0.402 for 137 residues, respectively. The different contact regions of the nanobodies to eDHFR are indicated by dashed circles. The nanobody-DHFR complexes lack small molecular ligands at the DHFR active site, indicating that the observed DHFR conformations are stabilized by the nanobodies.

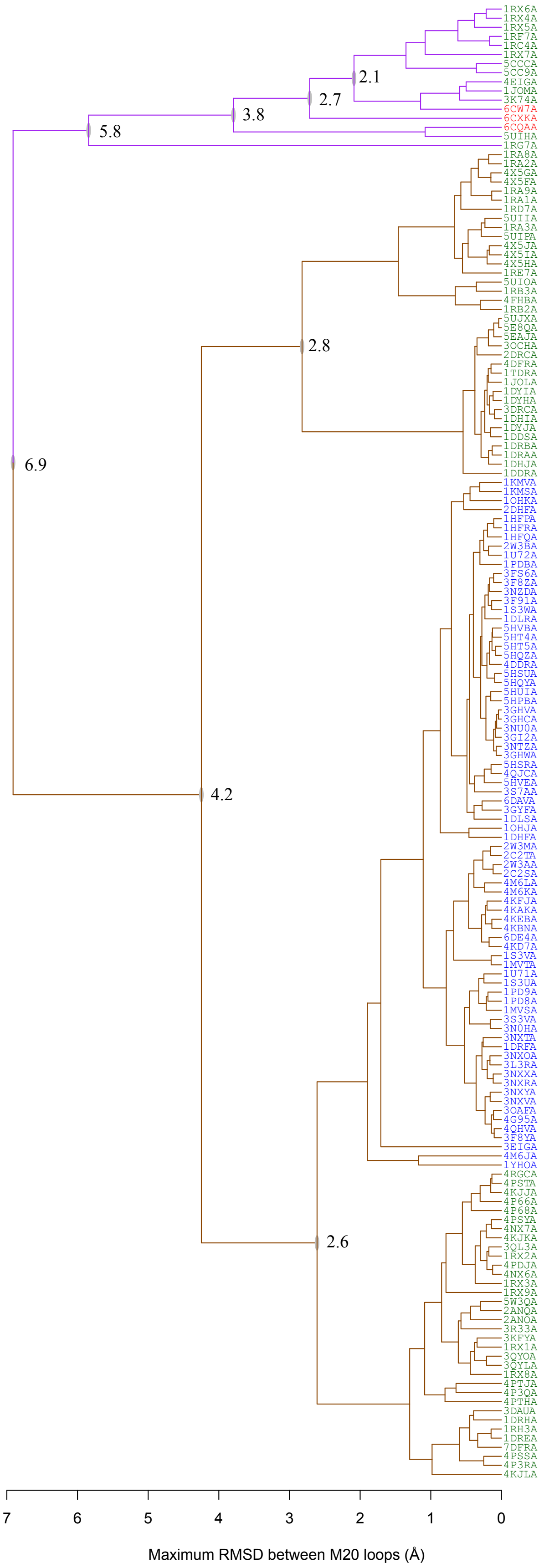
6.9
5.8
3.8
2.7
2.1
4.2
2.8
2.6
1RX6A
1RX4A
1RX5A
1RF7A
1RC4A
1RX7A
5CCCA
5CC9A
4EIGA
1JOMA
3K74A
6CW7A
6CXKA
6CQAA
5UIHA
1RG7A
1RA8A
1RA2A
4X5GA
4X5FA
1RA9A
1RA1A
1RD7A
5UIIA
1RA3A
5UIPA
4X5JA
4X5IA
4X5HA
1RE7A
5UIOA
1RB3A
4FHBA
1RB2A
5UJXA
5E8QA
5EAJA
3OCHA
2DRCA
4DFRA
1TDRA
1JOLA
1DYIA
1DYHA
3DRCA
1DHIA
1DYJA
1DDSA
1DRBA
1DRAA
1DHJA
1DDRA
1KMVA
1KMSA
1OHKA
2DHFA
1HFPA
1HFRA
1HFQA
2W3BA
1U72A
1PDBA
3FS6A
3F8ZA
3NZDA
3F91A
1S3WA
1DLRA
5HVBA
5HT4A
5HT5A
5HQZA
4DDRA
5HSUA
5HQYA
5HUIA
5HPBA
3GHVA
3GHCA
3NU0A
3GI2A
3NTZA
3GHWA
5HSRA
4QJCA
5HVEA
3S7AA
6DAVA
3GYFA
1DLSA
1OHJA
1DHFA
2W3MA
2C2TA
2W3AA
2C2SA
4M6LA
4M6KA
4KFJA
4KAKA
4KEBA
4KBNA
6DE4A
4KD7A
1S3VA
1MVTA
1U71A
1S3UA
1PD9A
1PD8A
1MVSA
3S3VA
3N0HA
3NXTA
1DRFA
3NXOA
3L3RA
3NXXA
3NXRA
3NXYA
3NXVA
3OAFA
4G95A
4QHVA
3F8YA
3EIGA
4M6JA
1YHOA
4RGCA
4PSTA
4KJJA
4P66A
4P68A
4PSYA
4NX7A
4KJKA
3QL3A
1RX2A
4PDJA
4NX6A
1RX3A
1RX9A
5W3QA
2ANQA
2ANOA
3R33A
3KFYA
1RX1A
3QYOA
3QYLA
1RX8A
4PTJA
4P3QA
4PTHA
3DAUA
1DRHA
1RH3A
1DREA
7DFRA
4PSSA
4P3RA
4KJLA
7
6
5
4
3
2
1
0
Maximum RMSD between M20 loops (Å)

Supplementary Fig. 3, related to Fig. 8. Hierarchical clustering of 162 DHFR structures based on the Cα RMSD of the Met20 loop. All structures fall in one of the two main clusters, the "closed" (brown) and "occluded" (purple) conformers. Structures from human, *E. coli* and this study are colored in blue, green and red, respectively, and labeled in the XXXXY format, where XXXX is the PDB code and Y is the PDB chain ID. If two branches are separated by more than 2 Å by their pairwise RMSD, the distance is labeled at the node where the two branches are joined.

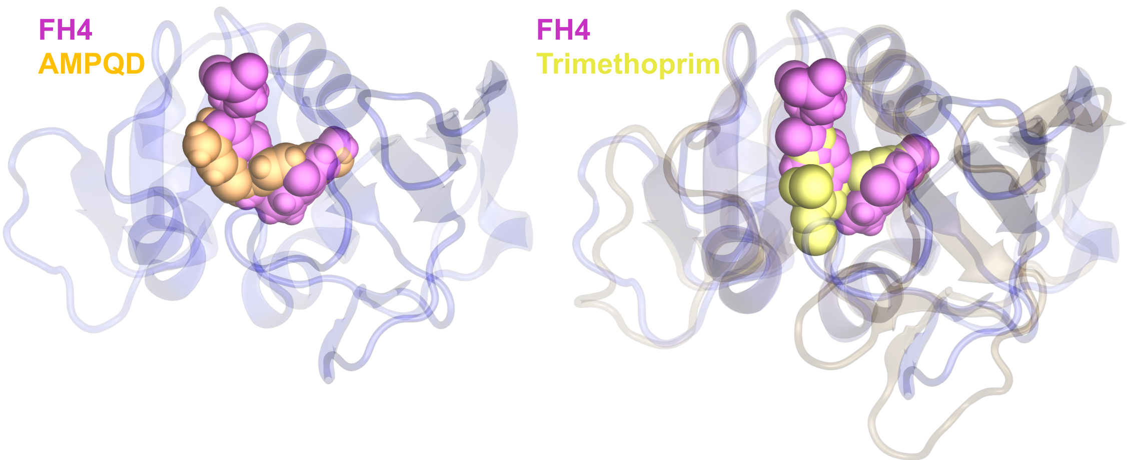

Supplementary Fig. 4, related to Fig. 9. Structural comparison of eDHFR:FH4 binary complex with inhibitory complexes. Left, eDHFR:FH4 (purple) and eDHFR:AMPQD (orange) complexes; Right, eDHFR:FH4 (purple) and *Mycobacterium tuberculosis* DHFR:Trimethoprim:NADPH complexes (yellow, PDB: 1DG5)[7]. The ligands are shown as van der Waal spheres with the protein secondary structures shown as transparent cartoons.

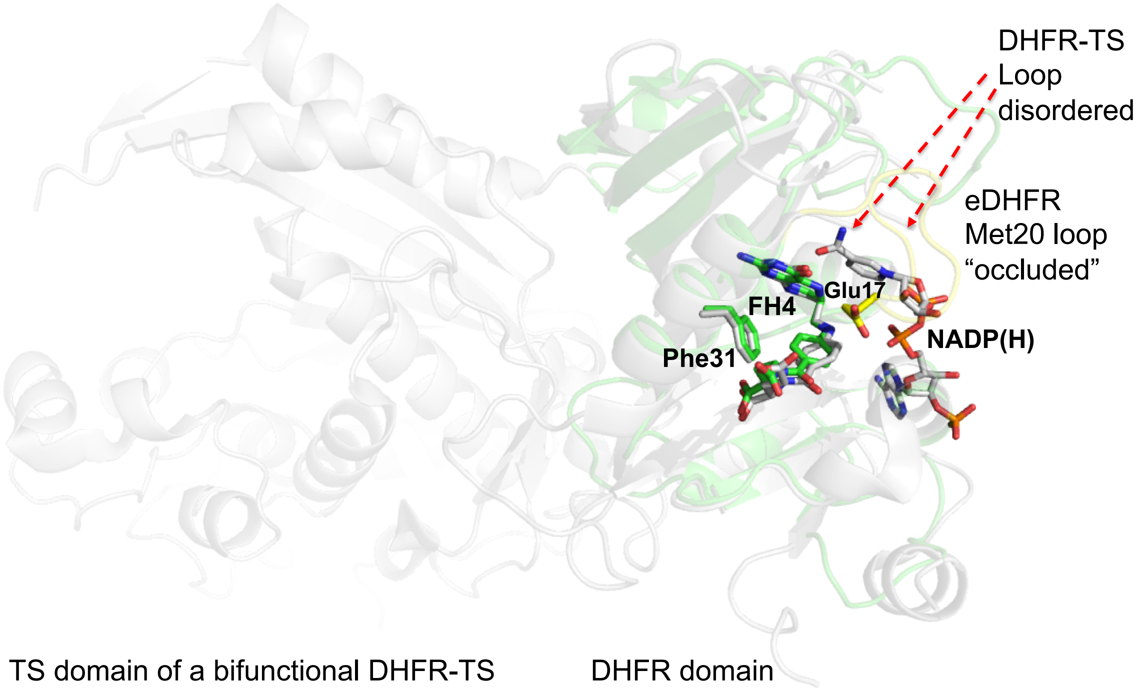

Supplementary Fig. 5. Structural comparison of the eDHFR:FH4 binary complex (green) and *Trypanosoma cruzi* bifunctional dihydrofolate reductase-thymidylate synthase ternary complex[6] with FH4 and $NADP^+$ (white). *T. cruzi* DHFR-TS contains an N-terminal DHFR domain and a C-terminal TS domain (PDB entry 5T7O)[6]. Only one subunit of the DHFR-TS homodimer (chain A) is shown despite both active sites are similar[6]. The protein secondary structures are shown as cartoons and ligands are shown as sticks. The structural alignment was carried out using PyMOL[5] based on the dihydrofolate reductase domains. The Met20 loop of eDHFR and Glu17 in proximity to the benzoyl ring FH4 are shown in yellow. In contrary, the corresponding loop was disordered in the *T. cruzi* DHFR-TS structure with its two ends indicated by red dashed arrows. The geometry of FH4 in both structures is similar with an obvious difference in the orientation of the benzoyl ring relative to the conserved Phe residue (Phe31 in eDHFR and Phe52 in *T. cruzi* DHFR-TS). The observed structural differences are consistent with the currently proposed slow release mechanism of the occluded eDHFR:FH4 complex and the role of favorable π-π interactions between the benzoyl ring of FH4 and conserved Phe31 as well as the Met20 in stabilizing the occluded product complex. In contrast to the occluded conformation of the eDHFR:FH4 complex, the bifunctional *T. cruzi* DHFR-TS ternary complex shows active site loop disorder. Furthermore, the nicotinamide ring region electron density was reported to be incomplete[6]. The *T. cruzi* DHFR-TS structure was obtained from a protein stock solution with 1mM NADPH and 1mM dUMP added prior to crystallization[6]. Thus, the possibility exists that the exogenous substrate and cofactor caused turnover of endogenous methylene tetrahydrofolate by DHFR-TS, although it was postulated that FH4 in the *T. cruzi* DHFR-TS structure could be co-purified[6]. Interestingly, a previous 2D NMR study estimated that ~5-7% of purified eDHFR corresponds to the eDHFR:$NADP^+$:FH4 ternary form[8]. The pairwise sequence identities between eDHFR, human DHFR and the *T. cruzi* bifunctional enzyme's DHFR domain are in a similar range of 31-35%.

Supplementary Table 1: Statistics for Data Collection and Refinement of the Crystal Structures of *E. coli* DHFR

Statistic	FH4 complex	FH2 Complex	Ternary Complex	AMPQD Complex
Protein Data Bank ID code	6CW7	6CXK	6CYV	6CQA
Space group	$P2_12_12_1$	$P2_12_12_1$	$P2_12_12_1$	$P6_12\ 2$
Cell dimensions				
a, b, c (Å)	33.9, 51.5, 77.8	33.7, 51.5, 77.4	34.9, 58.8, 79.3	64.7, 64.7, 215.7
α, β, γ (°)	90.0, 90.0, 90.0	90.0, 90.0, 90.0	90.0, 90.0, 90.0	90.0, 90.0, 120.0
Wavelength (Å)	0.97931	0.97931	0.97931	0.97931
Resolution of data collection (Å)	42.97 – 1.03 (1.09-1.03)	42.89 – 1.11 (1.18-1.11)	32.87– 1.30 (1.35-1.30)	34.17 – 2.20 (2.28-2.20)
No. of unique reflections	65923 (9115)	53268 (7579)	40813 (3991)	14200 (1236)
Completeness % (Å)	97.3 (84.2)	97.9 (87.3)	99.8 (99.5)	98.5 (89.4)
Redundancy	6.4 (2.8)	6.3 (2.8)	7.1 (6.3)	16.6 (6.8)
R_{sym}[a]	0.064 (1.02)	0.067 (0.86)	0.085 (2.11)	0.123 (2.93)
$CC_{1/2}$[b]	0.999 (0.471)	0.999 (0.571)	0.999 (0.307)	0.999 (0.377)
CC*[b]	1.00 (0.80)	1.00 (0.85)	1.00 (0.69)	1.00 (0.74)
I/σ[c]	11.3 (0.7)	13.4 (1.2)	12.3 (0.9)	19.8 (0.7)
Resolution range in refinement (Å)	31.06 – 1.03 (1.07-1.03)	30.95 – 1.11 (1.15-1.11)	32.87 – 1.30 (1.35-1.30)	34.17 – 2.20 (2.28-2.20)
No. of unique reflections (work/test)	65896/1995	53202/1993	40798/1995	13920/2455
R_{work}[d]	0.186 (0.361)	0.179 (0.382)	0.176 (0.343)	0.223 (0.470)
R_{free}[e]	0.206 (0.349)	0.196 (0.404)	0.204 (0.347)	0.259 (0.486)
Mean coordinate error[f] (Å)	0.13	0.14	0.16	0.40
Rmsd bond length (Å)	0.008	0.009	0.013	0.006
Rmsd bond angles (°)	1.39	1.33	1.52	1.01
Average B value ($Å^2$) (overall/protein/water/ligand)	16.4/14.7/26.3/24.9	18.6/17.0/28.6/25.4	23.8/21.7/34.7/26.3	69.6/69.9/65.5/63.9
No. of non-hydrogen atoms	1672	1637	1614	1339
No. of protein atoms	1428	1394	1293	1267
No. of water atoms	205	202	241	39
No. of ligands and ions	1 FH4, 4 Mg^{2+}, 3 Cl^-	1 FH2, 5 Mg^{2+}, 1 Cl^-	1 FH2, 1 NAD^+	2 SO_4^{2-}, 1AMPQD
Ramachandran statistics[g] (%)	98.3, 1.7, 0.0	98.2, 1.8, 0.0	99.0, 1.0, 0.0	97.5, 2.5, 0.0

Values in parenthesis are for the highest resolution shell.

[a]$R_{sym} = \sum_{hkl}\sum_i |I_i(hkl) - \langle I(hkl)\rangle| / \sum_{hkl}\sum_i I_i(hkl)$, where $I_i(hkl)$ is the intensity of an individual measurement of the symmetry related reflection and $\langle I(hkl)\rangle$ is the mean intensity of the symmetry related reflections.

[b]$CC_{1/2}$ = percentage of correlation between intensities from random half-datasets. $CC_{1/2}$ above 0.1 is considered significant[1]. $CC^*=[2CC_{1/2}/(1+ CC_{1/2})]^{1/2}$. CC* estimates the value of CC_{true}. CC* (or $CC_{1/2}$) is a robust, statistically informative quantity useful for defining the high-resolution cutoff of diffraction data to improve model quality[1].

[c]I/σ is defined as the ratio of averaged value of the intensity to its standard deviation.

[d]$R_{work} = \sum_{hkl} ||F_{obs}| - |F_{calc}|| / \sum_{hkl} |F_{obs}|$, where F_{obs} and F_{calc} are the observed and calculated structure-factor amplitudes.

[e]R_{free} was calculated as R_{cryst} using randomly selected small fractions (typically <10%) of unique reflections that were omitted from the structure refinement.

[f]Mean coordinate error was calculated based on maximum likelihood.

[g]Ramachandran statistics indicate the percentage of residues in the most favored, additionally allowed and outlier regions of the Ramachandran diagram as defined by MolProbity[2]

Supplementary References

1. Karplus, P. A. & Diederichs, K. Linking crystallographic model and data quality. *Science* **336**, 1030-1033 (2012).
2. Chen, V. B., et al. MolProbity: all-atom structure validation for macromolecular crystallography. *Acta Crystallogr. D Biol. Crystallogr.* **66**, 12-21 (2010).
3. Oyen, D., Srinivasan, V., Steyaert, J. & Barlow, J. N. Constraining enzyme conformational change by an antibody leads to hyperbolic inhibition. *J. Mol. Biol.* **407**, 138-148 (2011).
4. Oyen, D., Wechselberger, R., Srinivasan, V., Steyaert, J. & Barlow, J. N. Mechanistic analysis of allosteric and non-allosteric effects arising from nanobody binding to two epitopes of the dihydrofolate reductase of *Escherichia coli. Biochim. Biophys. Acta.* **1834**, 2147-2157 (2013).
5. The PyMOL Molecular Graphics System, Version 2.0 Schrödinger, LLC.
6. Panecka-Hofman, J., et al. Comparative mapping of on-targets and off-targets for the discovery of anti-trypanosomatid folate pathway inhibitors. *Biochim. Biophys. Acta. Gen. Subj.* **1861**, 3215-3230 (2017).
7. Li, R., Sirawaraporn, R., Chitnumsub, P., Sirawaraporn, W., Wooden, J., Athappilly, F., Turley, S. & Hol, W. G. J. Three-dimensional structure of M. tuberculosis dihydrofolate reductase reveals opportunities for the design of novel tuberculosis drugs. *J. Mol. Biol.* **295**, 307-323 (2000).
8. Bhabha, G., Tuttle, L., Martinez-Yamout, M. A. & Wright, P. E. Identification of endogenous ligands bound to bacterially expressed human and *E. coli* dihydrofolate reductase by 2D NMR. *FEBS. Lett.* **585**, 3528-3532 (2011).
